# Phylogenetic Networks and Parameters Inferred from HIV Nucleotide Sequences of High-Risk and General Population Groups in Uganda: Implications for Epidemic Control

**DOI:** 10.3390/v13060970

**Published:** 2021-05-24

**Authors:** Nicholas Bbosa, Deogratius Ssemwanga, Rebecca N. Nsubuga, Noah Kiwanuka, Bernard S. Bagaya, John M. Kitayimbwa, Alfred Ssekagiri, Gonzalo Yebra, Pontiano Kaleebu, Andrew Leigh-Brown

**Affiliations:** 1 Medical Research Council (MRC)/Uganda Virus Research Institute (UVRI) and London School of Hygiene and Tropical Medicine (LSHTM) Uganda Research Unit, Entebbe 256, Uganda; Deogratius.Ssemwanga@mrcuganda.org (D.S.); nrebeccansubuga@gmail.com (R.N.N.); Pontiano.Kaleebu@mrcuganda.org (P.K.); 2 Department of General Virology, Uganda Virus Research Institute, Entebbe 256, Uganda; assekagiri@gmail.com; 3 School of Public Health, College of Health Sciences, Makerere University, Kampala 256, Uganda; nkiwanuka@gmail.com; 4 IAVI-UVRI HIV Vaccine Program, Entebbe 256, Uganda; bagayabs@yahoo.com; 5 Department of Immunology and Molecular Biology, College of Health Sciences, Makerere University, Kampala 256, Uganda; 6 Centre for Computational Biology, Uganda Christian University, Mukono 256, Uganda; kittsra@gmail.com; 7 The Roslin Institute, Royal (Dick) School of Veterinary Medicine, Easter Bush Campus, University of Edinburgh, Edinburgh EH25 9RG, UK; Gonzalo.Yebra@ed.ac.uk; 8 Institute of Evolutionary Biology, University of Edinburgh, Edinburgh EH9 3FL, UK; A.Leigh-Brown@ed.ac.uk

**Keywords:** HIV, phylogenetic, transmission network, parameters, phylodynamic, model, populations, epidemic control, prevention

## Abstract

Phylogenetic inference is useful in characterising HIV transmission networks and assessing where prevention is likely to have the greatest impact. However, estimating parameters that influence the network structure is still scarce, but important in evaluating determinants of HIV spread. We analyzed 2017 HIV *pol* sequences (728 Lake Victoria fisherfolk communities (FFCs), 592 female sex workers (FSWs) and 697 general population (GP)) to identify transmission networks on Maximum Likelihood (ML) phylogenetic trees and refined them using time-resolved phylogenies. Network generative models were fitted to the observed degree distributions and network parameters, and corrected Akaike Information Criteria and Bayesian Information Criteria values were estimated. 347 (17.2%) HIV sequences were linked on ML trees (maximum genetic distance ≤4.5%, ≥95% bootstrap support) and, of these, 303 (86.7%) that consisted of pure A1 (n = 168) and D (n = 135) subtypes were analyzed in BEAST v1.8.4. The majority of networks (at least 40%) were found at a time depth of ≤5 years. The waring and yule models fitted best networks of FFCs and FSWs respectively while the negative binomial model fitted best networks in the GP. The network structure in the HIV-hyperendemic FFCs is likely to be scale-free and shaped by preferential attachment, in contrast to the GP. The findings support the targeting of interventions for FFCs in a timely manner for effective epidemic control. Interventions ought to be tailored according to the dynamics of the HIV epidemic in the target population and understanding the network structure is critical in ensuring the success of HIV prevention programs.

## 1. Introduction

The HIV epidemic in Uganda is heterogeneous consisting of concentrated sub-epidemics within a generalized one [1]. Key populations that include long-distance truckers, female sex workers (FSWs), men who have sex with men (MSM), people who inject drugs, uniformed forces and fisherfolk are disproportionately affected by HIV relative to the general population (GP) [2,3,4,5,6]. Fisherfolk and FSWs have the highest HIV incidence rates among key populations, estimated at 6 per 100 person-years at risk (PYAR) [7] and 3 per 100 PYAR [3,8,9], respectively. These figures are significantly higher than the estimated rate of less than 1/100 PYAR in the general population (GP) [10]. In this study, the term fisherfolk communities (FFCs) refers to groups of people living in villages located along the shores of Lake Victoria or on islands and who are largely dependent on the harvest or processing of fishery resources [5]. In contrast, the GP refers to persons living in communities that are adjacent to the FFCs (inland settlements approximately 10−40 km distance) who are not primarily dependent on fishing-related activities but are mostly agrarian or involved in trading [7,11]. Female sex workers include persons involved in either commercial or transactional sex [1,5,8].

Phylogenetic analyses that mostly rely on molecular sequence data to make inferences have become an important method to characterise HIV transmission networks [11,12]. These approaches have provided useful insights in understanding HIV transmission and prevention [13,14,15,16,17]. In Uganda, phylogenetic-based studies at the MRC/UVRI & LSHTM Uganda Research Unit and Rakai Health Sciences Program among key populations showed that HIV-hyperendemic FFCs are mostly recipients of HIV from the neighboring general population [11,12]. These findings were corroborated by a recent study which revealed preferential migration of high-risk persons into the FFCs with significantly higher HIV prevalence [18]. This could imply that targeted interventions in these high HIV-prevalence and incidence communities alone would not be likely to control the HIV epidemic in the neighboring general populations. Such studies have highlighted the role of phylogenetic analyses in identifying groups that are at the highest risk of acquiring HIV infection and evaluating where prevention is likely to succeed. In combination with socio-demographic or epidemiological data, phylogenetic analyses have been applied to identify traits associated with onward HIV transmission and groups that are at highest risk of acquiring or passing on HIV in key and general populations [19,20]. Phylogenetic based studies have reported concentrated sub epidemics involving high-risk groups in Uganda [13,21]. Nonetheless, extra-community viral transmissions have also been found to contribute to HIV spread in rural populations [17] and high levels of sexual mixing between partners in FFCs, FSWs and the GP have been documented within our cohorts [19,22,23]

Although HIV transmission network studies have provided useful insights in understanding the underlying dynamics of viral spread in different populations [14,24], estimating parameters that influence the network structure or formation [25,26] is still uncommon. This is critical in understanding HIV spread and effective epidemic control in populations. For example, within the UK’s MSM population, it was shown that random interventions were unlikely to be effective in controlling HIV epidemics in networks that are defined by a preferential association process [27]. The structure of HIV transmission networks underlying an epidemic could greatly influence the rate of disease spread and epidemic growth [28], directly impacting on the effectiveness of interventions [29]. For instance, a study that evaluated the effect of network structures on vaccination strategies showed that the structure of the network had a more profound impact on disease spread and incidence than the vaccination strategy [30]. In the study presented here, we used phylogenetic-based analyses supported by mathematical models to test the hypothesis that the network structure in key populations was scale-free and to make predictions of how the network structure could influence effective HIV epidemic control.

## 2. Materials and Methods

### 2.1. Study Design and Population

Cross-sectional surveys were carried out in FFCs of Lake Victoria, FSWs and GP groups between 2009 and 2016. The study was nested in the MRC/UVRI and LSHTM Uganda Research Unit Molecular Epidemiology study that aimed to determine viral subtypes and transmission linkages in both high-risk and general population groups in Uganda. HIV partial *pol* sequences (n = 2017) from the three populations (FFCs, FSWs and GP) were analysed by phylogenetic methods. Sequences (n = 728) from the FFCs included: the HIV Combination Intervention (HIVCOMB) [31] (n = 365), Masaka [32] (n = 210), The Lake Victoria Island Intervention Study on Worms and Allergy-Related diseases (LaVIISWA) [33,34] (n = 110) and a cohort of recently infected individuals (estimated sero-conversion of 6 months) [22] (n = 43). Sequences (n = 592) from FSWs included those from the good health for women’s project (GHWP) [3,8,9] that comprised women above 18 years of age, involved in commercial sex work and/or high-risk sexual behavior in Kampala. Additionally, HIV sequences from the GP (n = 697) were obtained from individuals receiving care at health centres neighbouring FFCs and FSWs hotspots including those diagnosed as HIV positive during counselling and testing (VCTs) in the districts of Kampala, Mpigi and Kalungu. After obtaining written informed consent, 10 mL of blood were collected by venepuncture from the study participants including those on antiretroviral therapy. The study inclusion criteria involved recruitment of HIV positive individuals above 18 years of age in the FFCs and FSWs and at least 16 years in the GP. To avoid breaching study participant confidentiality, the precise identity of the study sites was not shown, because the fisherfolk lived in relatively small fishing villages while the FSWs operated mostly in the same communities in which they could be identified.

### 2.2. HIV DNA Sequencing and Sequence Editing

HIV DNA sequencing was performed at the MRC/UVRI and LSHTM Basic Science Virology Laboratories (Entebbe, Uganda) that are accredited as a regional centre for HIV drug resistance genotyping by the World Health Organization (WHO). Briefly, pro-viral DNA was extracted from cell pellets using the QIAamp Viral DNA kit (Qiagen, Hilden, Germany) to increase the amplification and sequencing success rate in samples from low-viraemic patients. Nested PCR was then performed to amplify the HIV *pol* region (protease codon 1-99 and the amino terminus of reverse transcriptase codons 1-320) using gene specific primers as described elsewhere [22]. HIV DNA genotyping of the amplified fragments was performed using the Big Dye Terminator v3.1 Cycle Sequencing Kit (Applied Biosystems) and results were analyzed using the ABI 3130 Genetic Analyzer (Applied Biosystems, Foster City, CA, USA) [22]. Raw sequence data was edited using the Sequencher v4.10.1 (Gene codes Corporation, Ann Arbor, MI, USA) and RECall [35] software. Multiple sequence alignments were performed using MAFFT [36], edited and trimmed to equal length (1257 bp) in Geneious v9.0.5 [37]. HIV drug resistance mutations sites as identified in the Stanford University HIV drug resistance database [38] were removed to minimize bias due to convergent evolution [39,40]. Duplicate sequences were also removed using the ElimDupes program [41] to ensure that only one sequence per individual was included in the dataset prior to the phylogenetic analysis.

### 2.3. Phylogenetic Analysis

Maximum likelihood (ML) phylogenetic trees were constructed using the randomized Accelerated Maximum Likelihood (RAxML) program [42] with a general time reversible (GTR) model of nucleotide substitution and determined as the fittest model by the Akaike Information Criteria (AIC) in Jmodeltest [43]. Potentially linked HIV sequences were identified on the ML trees using the Cluster Picker program [44] at a maximum genetic distance (GD) distance of 4.5% with high bootstrap support (≥0.95). Results were viewed in FigTree v1.4.2 [45]. All sequences were assigned unique IDs to anonymize the study participants and delink them from any clinical identifiers.

### 2.4. HIV Subtyping and Bayesian Phylogenetic Inference in BEAST v1.8.4

HIV sequences were classified using COMET [46], SCUEAL [47] and REGAv3 [48] as previously described [12] to determine the predominant circulating strains. To identify HIV sequences with high evolutionary rates and whose genetic divergence was incongruent with their sampling times, we analyzed the dataset in TempEst v1.5 [49]. To improve the temporal signal for the BEAST analysis and the likelihood of MCMC chains’ convergence, we included historical sequences that were sampled in the 1980s during the early years of the HIV epidemic in Uganda [21]. Sequences classified as pure A1 and D subtypes were analyzed in BEAST [50] and a Bayesian Markov Chain Monte Carlo (MCMC) method was implemented in BEAST v1.8.4 for 300 million generations sampling after every 10,000th iteration. We used an uncorrelated lognormal-distributed relaxed molecular clock with the SRD06 model of nucleotide substitution [51] and a coalescent skygrid model. Marginal likelihood estimates of different model combinations were compared using the path sampling/stepping-stone method [52] to determine models that best fitted the data. An evolutionary rate of 1.5 × 10^−3^ substitutions/site/year was expected based on estimates from our previous study [21]. A lognormal prior distribution was specified for the evolutionary rate mean (ucld.mean; initial value = 1, mean = 0 and stdev = 1.0) and a normal prior distribution for the evolutionary rate standard deviation (ucld.stdev; initial value = 0.3, mean = 0.3 and stdev = 1.0). Two independent BEAST runs were combined using Log combiner [50] and convergence of the MCMC results was analysed in Tracer [53] based on the effective sample size (ESS) of parameter estimates after a 10% burn-in. Maximum Clade Credibility (MCC) trees were generated with Tree Annotator [54] to summarise the posterior tree distributions.

### 2.5. Phylodynamic Analysis and Network Generation

A time depth (TD) defined as the difference between the date in years of the most recent sample in a cluster and the time to the most recent common ancestor (TMRCA) was estimated from the MCC trees. This provided an approximation of the likely time of viral transmission between clusters [12,27]. This Bayesian approach improved the accuracy of the estimation of viral phylogenies that represented transmission networks. We used customized R [55] scripts to generate adjacency matrices of the networks for subsequent analysis.

### 2.6. Assessing for Power Law Distributions and Estimating Network Parameters

Certain networks have been reported to follow a power-law distribution [56] that is defined by a probability density function (PDF), $f\left(k\right),$ in which the frequency, $f\left(k\right),$ of an event is correlated to the size of that event, k, by the formula $f\left(k\right)=ck^{\gamma }$ where c and γ are constants [57]. In scale-free networks, considered to follow a power law distribution, the distribution of nodes is such that there exists very few, but highly connected, nodes in the network and very many nodes with low connectivity. In this case, the distribution has no peak and the long tail of the distribution is predicted to stretch with no scale, hence the term “scale-free”. Furthermore, the value for the exponent γ lies between 2 to 3 [58] and in such networks the spread of a disease will persist with no epidemic threshold [27,29]. In this study, the nodes in the network represented HIV infected individuals while the edges represented connections between nodes that correspond to sexual contacts or potential viral transmission events. The degree of a node was defined as the total number of edges attached to that node while the degree distribution was the frequency at which nodes with a given number of connections appeared in a network [29]. The poweRlaw package [59] implemented in the R software was used to fit a discrete power law distribution to our observed network degree distribution and estimate parameters for $k_{min}$ and γ. The $k_{min}$ defined as the minimum threshold for the degrees of a power law distribution was estimated using the Kolmogorov-Smirnov method [60] and was used to estimate values for the exponent (γ). We used a bootstrap resampling of 5000 iterations to test for the robustness of power law fit and generated *p*-values whereby, if *p* ≃ 0, then the model did not provide a reasonable fit to the data [59].

### 2.7. Model Fitting

We used the statnet package in R [61] to fit models that included the discrete pareto, yule, waring, negative binomial and the Poisson lognormal [62] to the observed degree distribution of our network data. Among the models, the discrete pareto, yule and waring models follow a power law distribution. However, the yule and waring models arise from a preferential attachment process where a new individual who joins the network is more likely to link to a high-degree node than a node with fewer links. The waring has been described as a natural generalisation of the yule model [63] and, while the process of network formation is similar for both models, the Waring additionally makes provision for the probability of non-preferential associations being a separate parameter from that which determines the preferential attachment process [64]. The negative binomial and Poisson lognormal models make the assumption that individuals have a fixed rate of linkage in the network over time [62]. We used the degreenet package within the statnet social network analysis suite of packages to perform 1000 bootstrap replicates for the model fitting. Model fit to the degree distribution were assessed using goodness of fit statistics that included the corrected Akaike Information Criteria (AICc) and Bayesian Information Criterion (BIC) scores. The model with the lowest AICc and BIC statistic was considered the best-fitting model.

## 3. Results

### 3.1. Network Generation

**Figure 1:**
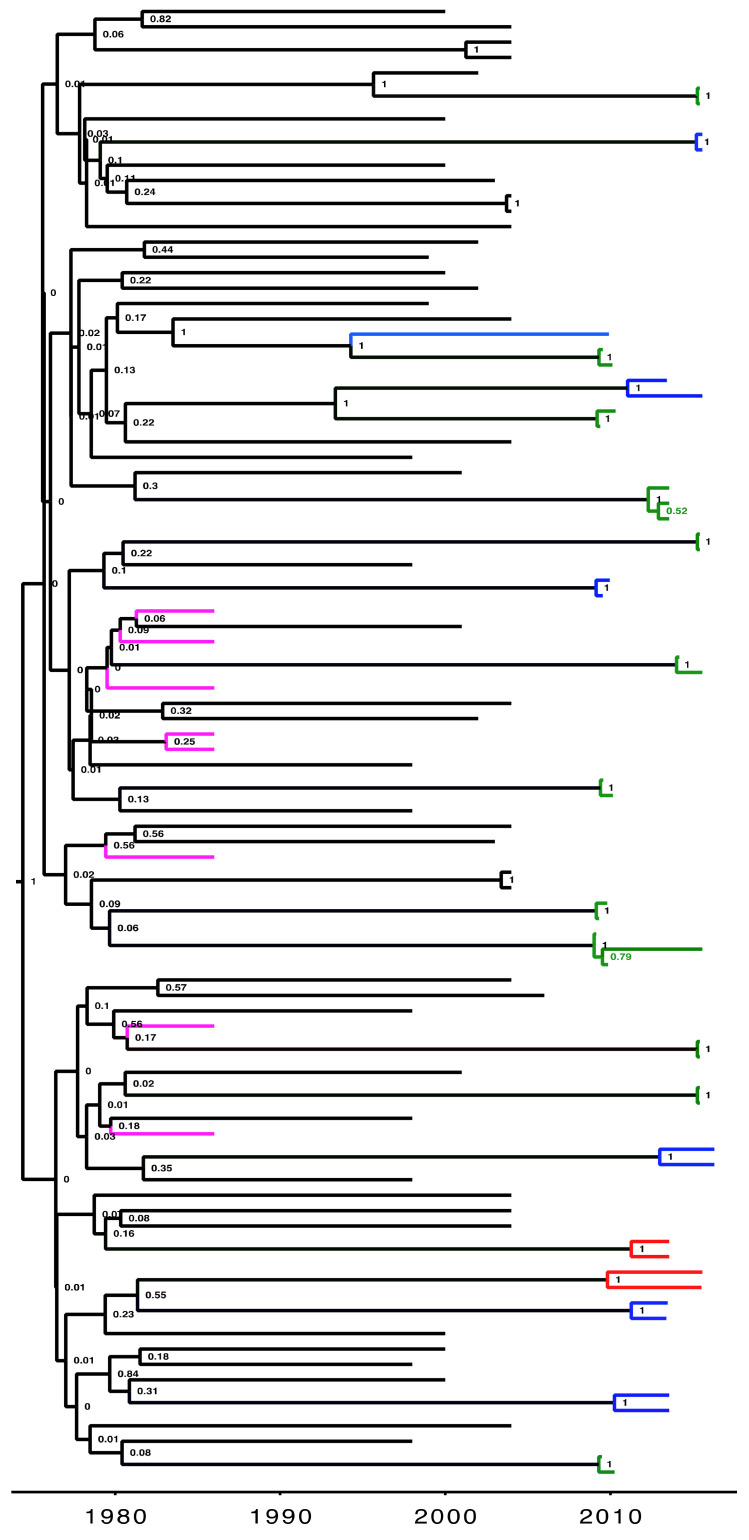
An example of a Maximum Clade Credibility (MCC) time-resolved phylogeny for HIV-1 sequences linked at a maximum genetic distance of 4.5%. Tips (without labels) on the tree represent sampled sequences that are linked (nodes supported by a high posterior probability of 1) with the branches colored according to the population (green, fisherfolk communities; red, female sex workers; blue, general population; purple, historical Ugandan samples collected during the early years (1980s) of the epidemic). The black colored branches are reference sequences that were downloaded from the Los Alamos HIV sequence database. Time scale at the bottom is in calendar years.

A total of 347 (17.2%) HIV sequences were linked in ML phylogenetic trees at a maximum pairwise genetic distance of 4.5% (>95% bootstrap support) of which 266 (76.7%) were linked to one other, 63 (18.2%) to two others, 12 (3.5%) to three others and six (1.7%) to five others. Of these, 303 (86.7%) that consisted of pure A1 (n = 168) and D (n = 135) subtypes were analyzed in BEAST v1.8.4 (44 excluded as inter-subtype recombinants) to generate time-calibrated phylogenetic trees (Figure 1). Table 1 below shows the distribution of sequences according to cluster size and time depth (TD) determined from a phylodynamic analysis. The TD in years for clusters/pairs provided an estimation to the time of HIV transmission by specifying the time to the last common ancestor of the viral strains in the transmitter [12,27].

At a TD of ≤5 years, 141 (46.5%) sequences were linked, of which 37 (26.2%) had a TD of ≤1 year, 31 (22%) a TD of 1−3 years and 73 (51.8%) a TD of 3−5 years. Thirty-seven (12.2%) individuals had a TD of 5−10 years, 110 (36.3%) had a TD of 10−20 years and 15 (5%) had a TD of 20−25 years. The majority of reconstructed HIV transmission networks were found at a time depth of between 1−5 years. Viral sequences with a TD of ≤5 years were assumed with a higher degree of certainty to be linked through viral transmission events. This threshold was used based on the assumption that any two individuals in a viral transmission network are linked if their nucleotide sequences are predicted to have diverged 5 years before the most recent sampled sequence [27].

#### Cluster Size Distribution and Assortativity Coefficient

At a time depth of ≤5 years, 141 HIV linked sequences from individuals in the different populations fell into 63 clusters (ranging from 2−6). Table 2 shows the frequency of clusters by size according to the study population. We computed the assortativity coefficients at the different cluster sizes with respect to population using the assortativity-nominal function of the igraph R package v1.2.6 [65].

The network comprised of 63 clusters of which 53 (84.1%) were dyads (nodes lined only to one other), 7 (11.1%) comprised of three individuals, two (3.2%) comprised of four individuals and one (1.6%) had six individuals. Among the 141 persons in the 63 clusters, the assortativity coefficient r for population or sampling region was 0.69 indicating assortative mixing across study locations. At the different cluster sizes, there was assortative mixing for sampling region with the exception of cluster size 6 which was non-assortative (r = −0.2).

### 3.2. Network Parameters

We used the poweRlaw package in R to generate network degree distributions and determine a power law fit [59,60]. At a TD of ≤5 years, the cluster size distribution followed a heavy-tailed distribution with a higher frequency of dyads and fewer higher degree nodes. We obtained a p value with a bootstrap resampling of 5000 iterations to test for a power law fit of our data. Typically, low p values are considered to be “good” because they suggest that the null hypothesis is unlikely to be correct. However, we applied the p value as a measure of the hypothesis we set out to verify and high values for the p value were considered acceptable. Consequently, if the value for p was large (close to 1), then the difference between the empirical data and the model can exclusively be due to statistical variations; otherwise, if it is small (p ≃ 0), then the model is not a plausible fit to the data [59,60]. A p = 0.75 (95% C.I 0.73−0.76) was obtained indicating a good fit for a power law distribution. This was obtained by comparing the empirical data to the model data to get an empirical distance followed by generating synthetic distances from parameters previously obtained for the γ and $k_{min}$ at each of the several iterations. The p value was then determined as a fraction of the synthetic distance that is larger than the empirical distance [60].

**Figure 2:**
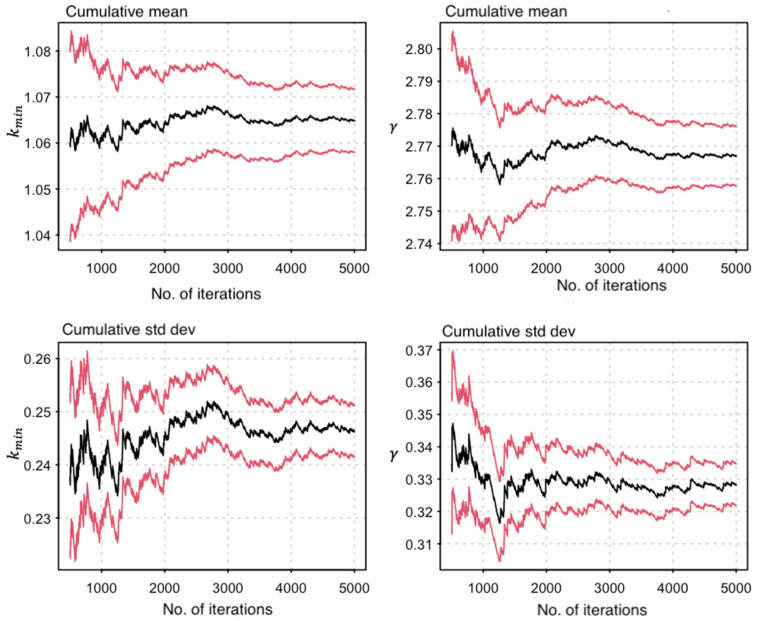
A graph showing the bootstrap resampling of parameter estimates. Panels show the cumulative mean and standard deviation (SD) of γ and $k_{min}$, respectively. In both panels, the vertical scale represents the parameter estimates and the horizontal scale represents the number of bootstrap iterations while the black and the red lines represent the mean and 95% CI intervals, respectively.

The gamma (γ) parameter was an estimation of the exponent for the power law distribution [57]. We obtained values for γ, $k_{min}$ and standard deviations (SD) from the network degree distribution and performed a bootstrap resampling of 5000 iterations to assess for parameter uncertainty (Figure 2). A mean (μ) value of 2.77 was obtained for γ with a 95% C.I (2.76−2.78) (Figure 2).

### 3.3. Model Fitting to Degree Distributions

Five models that included the discrete pareto, yule, waring, negative binomial and Poisson lognormal [62] were fitted to the observed network degree distribution in each of the three populations (FFCs, FSWs and GP). In Uganda, FFCs and FSWs are key populations considered ‘high HIV-risk’ groups while the GP is a relatively ‘lower HIV-risk’ group [1,5]. However, in this study, the lower sampling proportion in the larger GP could give rise to a downward bias in the number of observed viral transmissions [66], so we analyzed data from groups within the GP that were sampled more densely. Thus, we focused our analysis in the GP on Kisenyi, a slum in central Kampala with an estimated population of 19,400 people [67]. At an estimated HIV prevalence of 6.9% in Kampala [10], approximately 1300 persons are expected to be living with the virus in Kisenyi. Four hundred and sixty-five HIV sequences were genotyped from this geographical area, representing a sampling proportion of 34.7% of the estimated number of HIV positive individuals. At a 95% confidence interval (margin of error = 0.05), an estimated sample size of at least 300 HIV positive individuals would be statistically adequate [68] for analysis in this cohort. The network parameter γ was estimated from network degree distributions for each population and was used to make inferences about the processes underlying the distributions [57]. Networks of FFCs had the strongest fit for a power law distribution with a γ of 2.38 (95% C.I 2.35−3.47), while networks of FSWs and GP showed a relatively poorer fit for a power law distribution as shown in Table 3.

**Figure 3:**
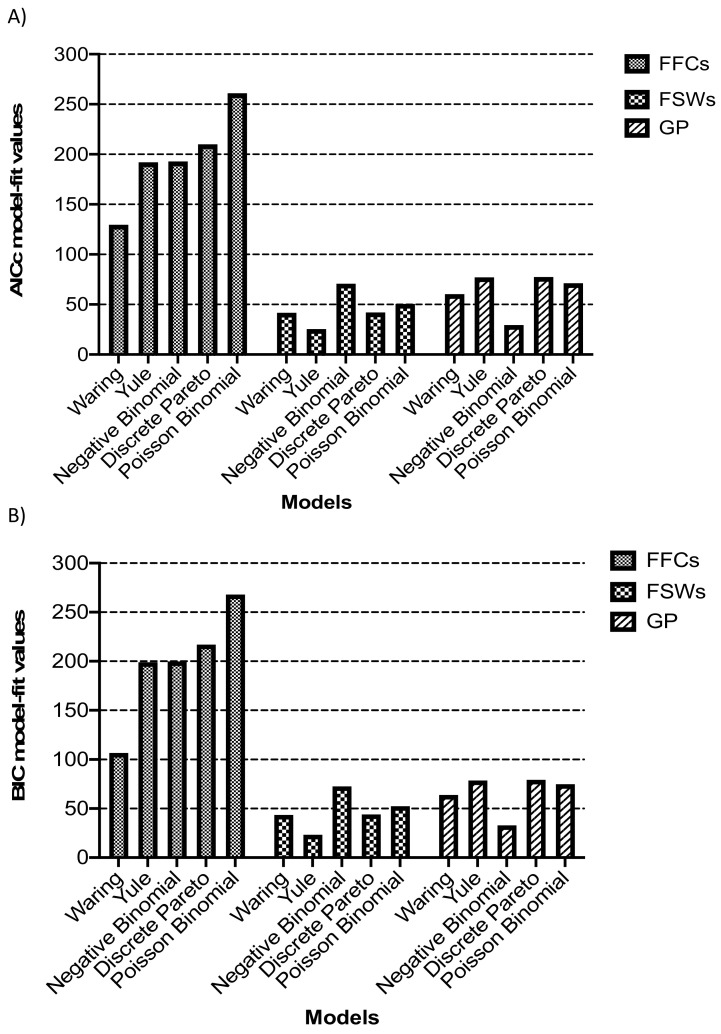
Model fit statistics. Five models that included the discrete Pareto, Yule, Waring, Negative Binomial and Poisson lognormal were fitted to the observed network degree distributions inferred from HIV sequence datasets of fisherfolk communities (FFCs), female sex workers (FSWs) and the general population (GP). (**A**) shows the corrected Akaike Information Criteria scores for the model fit while (**B**) shows the Bayesian Information Criteria (BIC) scores. The model with the lowest AICc and BIC scores was considered as the best-fitting model.

Fitting models to network degree distributions by population showed the waring, yule and negative binomial as the best fitting models in FFCs, FSWs and GP, respectively (Figure 3). We tested for differences in model fit between the waring, yule and negative binomial models using additional bootstrap resampling of 10,000 iterations in the three populations. Simulations revealed that the yule and negative binomial never fitted as well as the waring to the network data of FFCs (data not shown). Similarly, the negative binomial and yule models were the preferred better fitting models even with increased simulations for networks in the GP and FSWs.

In this analysis, model fit to network degree distributions involving high-risk populations that included in this case the FFCs and FSWs tended to lean towards preferential attachment models (waring and yule) in contrast to the general population.

## 4. Discussion

We analyzed HIV nucleotide sequences from three Ugandan populations that included fisherfolk communities (FFCs), female sex workers (FSWs) and the general population (GP) by phylogenetic and modeling methods to estimate transmission network parameters, characterize the network structure and predict the implications for epidemic control. In our study, the majority of HIV transmission networks were found at a time depth of less than 5 years and, whereas the network degree distribution for all sequences followed a power law distribution, analysis of the data by population showed that networks of FFCs were best fitted by a waring model, FSWs by a yule model and the GP by the negative binominal model. Degree distributions with a power law scaling have previously been reported in networks of sexual contacts in Zimbabwe [29], Burkina Faso [69] and Uganda (Rakai) [70], but this is the first time HIV transmission network parameters have been estimated in African populations across different risk groups. In this study, we revealed that the underlying network structure in the fisherfolk population was best described by the waring distribution and likely characterized by a preferential attachment process. The estimated parameter γ (exponent) was 2.38 in networks of FFCs. Scale-free networks typically follow a power law degree distribution with highly connected nodes that potentially grow by preferential association. In such a scenario where γ lies between 2 and 3, there is no epidemic threshold [29] and HIV transmission involving a few but highly connected individuals in a network could result in the significant spread and persistence of the disease irrespective of its transmissibility [70,71]. This implies that a randomly distributed intervention would not control the epidemic since scale-scale free networks are not susceptible to random attacks [72].

Preferential attachment in networks of FFCs could result from several social or economic factors/constraints that include high-risk sexual behavior and having multiple sexual partners [73,74,75], preferential migration of high-risk persons [18], income disparities that promote sex work [76] and other socio-economic factors [77]. In the UK where HIV transmission in networks of the high-risk MSM population has been found to occur by preferential attachment, randomly implemented interventions would be unlikely to stop the epidemic [27]. This is because epidemics in such populations are largely concentrated and driven by core groups [78] and targeted interventions are therefore preferred for effective epidemic control [27,29,64]. Although the network structure of FSWs was best fitted by the yule model, they could not be described as scale-free due to a poor estimation of the gamma network parameter, which is likely to have resulted from an insufficient number of linked sequences with higher-degree nodes from this group, owing to the lack of sampling of their clients. In contrast, networks from the GP were best fitted by the negative binomial model, an indication of a fixed rate of partner acquisition. A similar observation was made in a population of Ugandan women in Rakai district where the sexual contact network was best fitted by a negative binomial model but differed from the men’s population that was defined by a highly skewed distribution [64].

This study has some limitations. First, HIV partial *pol* sequences were used which could have underrepresented the reconstructed viral transmission networks. Near full length HIV genomes improve phylogenetic reconstructions and hence provide better sensitivity in identifying clusters in the inferred viral transmission networks. Secondly, the FFCs were sampled more intensely than the GP, which could have biased the observed number of reconstructed networks from the fisherfolk population. Thirdly, we analyzed an insufficient number of sequences from groups like the FSWs which could have influenced the interpretation of results for this population. Fourthly, due to logistical constraints, an assessment of the effect of other factors such as gender or age on network formation were not explored which could be the focus of our future studies. Lastly, phylogenetically inferred networks could represent an incomplete sample of the viral transmission network due to unsampled intermediaries [25]; hence the need for robust sampling designs [66]. However, in our study, we applied a combination of phylogenetic and modeling approaches to analyze the underlying HIV transmission network structures in different populations and examined how this relates to prevention.

## 5. Conclusions

This study provides the first estimation of the transmission network parameters of HIV sequences from key and general population groups in Uganda. The network degree distribution in key populations followed a heavy-tailed power law distribution. Furthermore, networks of FFCs were found to be likely scale-free and shaped by preferential attachment. This suggests that while generalised random interventions could be effective in preventing disease spread in the GP, the control of HIV epidemics in high-risk populations like the FFCs would necessitate the characterisation and targeting of core groups in networks in a timely manner. Our previous studies have shown that the FFCs are net recipients for HIV transmission flow from the neighboring GP [11,12,19], suggesting that the high prevalence and incidence are traits of individuals who are recruited in the FFCs rather than being acquired once there. In conclusion, fine-scale network structure analyses could provide further insights in predicting the progression of the HIV epidemic and how it can be effectively controlled.

## Figures and Tables

**Table 1:** Shows the distribution of linked HIV sequences according to cluster size and TD.

	Cluster Size				Total
TD (years)	2	3	4	6	
≤5	106	21	8	6	141
5−10	34	3	_	_	37
10−20	82	24	4	_	110
20−25	6	9	_	_	15
Total	228	57	12	6	303

Abbreviations: TD, Time Depth.

**Table 2:** Cluster Size according to population for networks generated at a TD of ≤5 years.

			Cluster Size			
		2	3	4	6	Total
Population						
FFCs		21	5	1	1	28
GP		15	--	--	--	15
FSWs		13	--	1	--	14
FFCs/GP		1	1	--	--	2
FFCs/FSWs		2	1	--	--	3
GP/FSWs		1	--	--	--	1
Total		53	7	2	1	63
Assortativity Coefficient		0.83	0.59	0.47	−0.2	0.69

Abbreviations: FFCs: Fisherfolk Communities; FSWs: Female Sex Workers; GP: General Population.

**Table 3:** Transmission network parameter values estimated per population.

Population	$k_{min}{\;}^{a}$	γ $(\mu ){\;}^{b}$	95% Confidence Intervals	No. of Bootstraps
FFCs	1	2.38	2.35−3.47	5000
FSWs	1	3.51	3.22−4.21	5000
GP	1	4.03	3.84−4.73	5000

Abbreviations: FFCs: Fisherfolk Communities; FSWs: Female Sex Workers; GP: General Population. ^a^ Parameter $k_{min}$ is the minimum threshold for the degrees of a power law distribution ^b^ Parameter γ is the scaling parameter for the power law distribution.

## Data Availability

Study nucleotide sequences are available from GenBank under accession numbers: MG434786-MG435152. Data related to adjacency matrices, generating network degree distributions, fitting power law distributions, estimating parameters from degree distributions, testing for power law fit, model fitting and generation of model fit statistics are available from the corresponding author on reasonable request.
